# Progress in Medicine

**Published:** 1899-08-26

**Authors:** 


### Progress in Medicine.

### FEVERS.

**Tetanus and Tetanus Antitoxin.**—Semple[1] states that the tetanus toxin travels to the nerve centres by (1) the blood stream, (2) along the nerves. The toxin has a special affinity for nerve substance, in which it is so securely fixed as to be unaffected by intravenous or hypodermic injection of antitoxin, which may, however, neutralise the toxin in the blood. Intracerebral injection was first recommended by Roux. Semple makes an incision over each temple, drills a hole through the skull with an Archimedian screw, and injects 2½ c.cm. of antitoxin into each frontal lobe. Hypodermic medication is still applied. The case he records recovered in the second week. Rambaud[2] gave intracerebral injections of antitoxin to 45 tetanised guinea pigs, and of these 35 recovered; of 17 treated with hypodermic injections only two survived. The antitoxin introduced into the brain does not cure the existing lesions, but prevents a spread of the disease. Death results in spite of treatment if the medulla has been implicated. He recommends in man that a dose of 5 or 6 c.cm be introduced slowly. A number of cases are quoted.

In a report on the treatment of tetanus by antitoxin,[3] it is stated that in attacks with an incubation period of 14 days or more, in which the temperature does not rise above 39 deg. C., and in which there is little dyspnœa, the results of serum therapy are favourable. In subacute cases Roux's method, if commenced early, yields good results; but in very acute cases the results are disappointing. Puerperal tetanus and tetanus neonatorum are not favourably affected. A list of 13 cases treated by Roux's method is appended; of these six died and seven recovered. A short abstract of a series of cases occurring in France, and treated by Roux's method, will be found in the *Quarterly Journal*, April, 1899. Kleine[4] reports two cases successfully treated by hypodermic injections. Both had a prolonged incubation period. In the first case, that of a man aged 50, the dose was 1,000 units; in the second case 400 units were injected, the patient being a boy of nine. In this case an urticarial rash appeared on the sixth day after the last injection. Church (in the New York *Medical Journal*, December 17th, 1898) records a case which was successfully treated conjointly by intracranial and intravenous injections of antitoxin. Chloral was also used. The symptoms which appeared 12 days after the injury rapidly improved. Forgue,[5] after unsuccessfully using chloral, bromide, and morphia, together with hypodermic injections of antitoxin, got immediate improvement from intracerebral injections. The incubation period in this case was 33 days, but the attack was of a most acute nature. Improvement followed the injection of 6 c.cm. into each frontal lobe in 10 hours, and recovery was complete in a week. Marshall,[6] in the course of nine days, injected 110 c.cm. of antitoxin hypodermically in the case of a child aged 12, in whom tetanic symptoms developed two weeks after a burn. There was no improvement until the fifth day of the treatment, but recovery was uninterrupted. An acute case of tetanus, in which antitoxin was of no avail, is recorded by Wace.[7] The incubation period was seven days, and death resulted on the second day of the illness. The spasms were never very marked, the chief symptoms being stiffness and retraction of the head. Twenty cubic centimetres were injected hypodermically on the first day. Collier,[8] in a subacute case having an incubation period of nine days, finding no improvement from chloral, morphia, or hypodermic injection of antitoxin, injected 10 c.cm. of Roux's serum into the subdural space over the cerebellum. Eighteen hours later there was remission of the spasm in the pharyngeal and cervical muscles. Recovery was slow, but complete. Lawrence and Hartley record a very similar case. The incubation period was here eight days. Bromide and chloral seemed at first to diminish the spasm, and carbolic acid certainly did so for a time. Hypodermic injections of antitoxin seemed useless, and rather to increase the spasms. Two and a half cubic centimetres of antitetanic serum were then injected into each frontal lobe. Next day the trismus was less marked, and patient felt more comfortable. Not until 10 days later did the spasms decrease, and recovery was not complete for three weeks. That the operation of intracerebral injection is not devoid of danger is well illustrated by a case reported by Gibb.[9] Two days after apparent convalescence cerebral symptoms were noticed, and the patent died 26 days later. The post-mortem examination showed abscesses in each frontal lobe surrounded by areas of deeply-congested tissue. Staphylococcus pyogenes aureus was cultivated from the pus, and, although every care was taken to secure asepsis at the time of drilling the hole for the injections, it seems certain that the infective agent must have been introduced with the serum.

**Typhoid Fever.**—The extreme variations which may occur in the incubation period of typhoid fever are well shown by some cases recorded by Atkinson in his report on the Kildwick epidemic. The exact date of the pollution of the water supply was known, and in a small community the several dates of invasion were easily ascertained. The period of incubation in some of the earlier cases could not have been more than 29 or less than 25 days. Of three children attending one school one sickened on the tenth and the others on the eleventh day. In one case the period could not have been more than eight days; whilst in another, in which the date could be fixed exactly, it was 18 days.

A case of typhoid fever without a lesion of the intestine is described by Bryant.[10] The patient, a male, aged one year and nine months, was suffering from broncho-pneumonia, together with diarrhœa and vomiting. There was high fever. The abdomen was distended, and the liver and spleen enlarged. The blood was tested shortly before death, and gave a positive Widal's reaction. At the post-mortem examination there was found septic broncho-pneumonia; no swelling, discoloration, or ulceration of Peyer's patches; the mesenteric glands were enlarged and acutely inflamed, and from these a pure culture of the typhoid bacillus was obtained. Bryant has collected reports of 15 similar cases, occurring at all ages. In 11 the characteristic signs and symptoms of typhoid fever were noted; enlargement of the spleen was present in eight, and of the mesenteric glands in nine. In all cases the specific bacillus was isolated from one or other organ. The presence of persistent diarrhœa in Bryant's case is of interest, showing as it does that this symptom is in no way dependent upon the extent or severity of intestinal lesions.

Restoski[11] in 346 cases of enteric fever found albuminuria in 59·2 per cent. In 37 out of these 205 cases, hyaline and epithelial casts showed the presence of nephritis. He gives details of two cases in which the earliest symptoms were those of acute nephritis, hæmaturia, albuminuria, and casts, but which subsequently developed into typical enteric fever, giving Widal's reaction. In his opinion every case of so-called idiopathic nephritis with high temperature should have the urine carefully tested for typhoid bacilli, and the blood examined for Widal's reaction. In this connexion Horton Smith[12] points out how much easier and more certain it is to demonstrate the characteristic bacillus in the urine of typhoid patients than in the fæces. Bacilli may be found in the fæces up till the beginning of the third week; after that time they rapidly disappear, unless the stools contain urine. Typhoid bacilli are present in the urine in 25 per cent of cases, and in 5 per cent. of cases they are present in such quantities as to render the urine turbid. This excretion of the bacilli in the urine extends far into convalescence. Although a trace of albumen is common the character of the urine is not as a rule altered. Their presence does not affect the prognosis. They are nearly always in pure culture. After the administration of urotropin, ten grains three times daily by the mouth, the bacilli frequently disappear from the urine, but this excretion should be regarded as infective until convalescence is established.

[1] Brit. Med. Jour., Jan. 7, 1899. [2] New York Med. Jour., Dec. 17, 1898. [3] University Med. Mag., Phil., Feb. 1899. [4] Duet. Med. Woch., Jan. 12, 1899. [5] Annals of Surgery, June, 1899. [6] Lancet, April 22, 1899. [7] Lancet, May 13, 1899. [8] Brit. Med. Jour., June 3, 1899. [9] Ibid., July 1, 1899. [10] Brit. Med. Jour., April 1, 1899. [11] Münch Med. Woch., Feb. 14, 1899. [12] Lancet, May 20, 1899.

